# Efficacy of Tuina combined with core stability training on lumbar stability and clinical outcomes in patients with lumbar disc herniation: a randomized controlled trial protocol

**DOI:** 10.3389/fmed.2026.1857731

**Published:** 2026-07-07

**Authors:** Xiaoyu Liang, Limei Huang, Huanzhen Zhang, Hongye Huang, Lechun Chen, Shuijin Chen, Jingjing Jiang, Zhigang Lin

**Affiliations:** 1 School of Acupuncture and Tuina, Fujian University of Traditional Chinese Medicine, Fuzhou, Fujian, China; 2 The Affiliated Rehabilitation Hospital, Fujian University of Traditional Chinese Medicine, Fuzhou, Fujian, China

**Keywords:** core stability training, lumbar disc herniation, musculoskeletal ultrasound, randomized controlled trial, rehabilitation, Tuina

## Abstract

**Background:**

Low back pain, radicular symptoms, and functional impairment are common manifestations of lumbar disc herniation (LDH), which can substantially impair patients' quality of life. Tuina therapy and core stability training are widely used conservative interventions for LDH, often applied as a combination of passive manual therapy and active exercise to alleviate symptoms and improve function. However, most existing clinical studies are limited by small sample sizes and methodological weaknesses, and robust evidence is lacking regarding whether the combined intervention is superior to either therapy alone.

**Methods:**

This study is a single-center, outcome assessor- and data analyst-blinded, randomized controlled trial. A total of 96 patients with LDH will be randomly allocated in a 1:1:1 ratio to the Tuina group, the core stability training group, or the combined intervention group. All participants will receive an 8-week intervention (three sessions per week), followed by a 1-month follow-up period. The primary outcome is the muscle activation ratio of the transversus abdominis assessed by musculoskeletal ultrasound. Secondary outcomes include imaging parameters, pressure biofeedback unit (PBU), the Visual Analog Scale (VAS), the Oswestry Disability Index (ODI), the Japanese Orthopedic Association (JOA) score, and the 36-Item Short Form Health Survey (SF-36). Objective outcomes (musculoskeletal ultrasound, MRI, X-ray, PBU) will be assessed by researchers blinded to group allocation. Patient-reported outcomes (VAS, ODI, JOA, and SF-36) are inherently unblinded as participants are aware of their treatment assignment. Statistical analysis will be performed without knowledge of group allocation.

**Conclusion:**

This study is expected to provide high-quality evidence on the efficacy and safety of Tuina combined with core stability training in improving lumbar stability and clinical outcomes in patients with LDH.

**Clinical trial registration:**

International Traditional Medicine Clinical Trial Registry (ITMCTR, http://itmctr.ccebtcm.org.cn/), identifier: ITMCTR2025001794.

## 1 Introduction

Lumbar disc herniation (LDH) is a common musculoskeletal disorder caused by degenerative changes in the intervertebral disc, resulting in protrusion or extrusion of the nucleus pulposus that compresses or irritates adjacent nerve roots. It typically presents with low back pain, radiating leg pain, and functional impairment (1). The prevalence of LDH in China ranges from 8% to 25%, making it one of the leading causes of low back pain (2). In recent years, the incidence of LDH has shown an increasing trend with a younger age of onset, most commonly between 30 and 55 years.Some studies suggest a higher incidence in men than in women. Approximately 95% of cases occur in the lower lumbar spine, particularly at the L4/5 and L5/S1 levels (3). Clinical manifestations range from isolated low back pain to severe neurological deficits such as cauda equina syndrome (4). These conditions substantially impair quality of life and impose a significant socioeconomic burden. Currently, the management of LDH can be broadly categorized into surgical and nonsurgical approaches. Given considerations of effectiveness and safety, nonsurgical treatments are generally recommended as the first-line option for most patients (5). Conservative strategies include pharmacological therapy, manual therapy, and exercise-based rehabilitation. Although medications can effectively relieve pain, they are often associated with adverse effects and have limited impact on restoring lumbar stability and functional capacity (6).

The pathogenesis of LDH is closely associated with intervertebral disc degeneration, reduced water content of the nucleus pulposus, narrowing of the intervertebral space, and decreased lumbar stability (7). Degenerative changes disrupt the biomechanical balance of the lumbar spine, leading to spinal instability (8). In turn, impaired stability further accelerates disc degeneration, forming a self-perpetuating cycle. In this context, the lumbar–abdominal musculature plays a critical compensatory role in maintaining spinal stability by distributing mechanical loads and supporting both static alignment and dynamic control (9). Therefore, restoring lumbar stability, particularly through improving neuromuscular function of the core musculature, has become a key therapeutic target in the conservative management of LDH (10).

Current clinical practice guidelines recommend Tuina therapy and exercise-based rehabilitation as important nonsurgical interventions for LDH (6, 11). Tuina, a traditional manual therapy, has been shown to relieve pain and improve functional outcomes, potentially through mechanisms such as enhancing local circulation, reducing inflammation, and modulating neural activity (12). The Tuina protocol applied in this study primarily targets the Dai meridian region, which anatomically corresponds to the lumbar–abdominal musculature and thoracolumbar fascia. Acupoints such as Daimai (GB26), Wushu (GB27), and Weidao (GB28) are anatomically located along the lateral abdominal wall, corresponding to the external oblique, internal oblique, and transversus abdominis muscle layers. These points have been widely selected in clinical and experimental studies based on their relevance to abdominal musculature and their role in modulating lumbar stability and core function (13).Previous studies from our research group have demonstrated that abdominal Tuina can increase transversus abdominis thickness and improve lumbar function (14, 15). However, existing evidence suggests that while Tuina is effective in alleviating pain and improving physical function, its effects are primarily limited to symptomatic relief, with insufficient influence on deep muscle activation and neuromuscular control, which are essential for long-term spinal stability (9, 16).

Core stability training, as a representative form of exercise-based rehabilitation, directly targets the neuromuscular mechanisms underlying spinal stability. It aims to improve coordination and activation of deep stabilizing muscles, thereby enhancing spinal and pelvic stability. By strengthening these muscles, core stability training can reduce excessive loading on superficial lumbar structures and address instability caused by muscle imbalance or fatigue (17, 18). Previous studies have demonstrated that core stability training can significantly reduce pain and improve functional performance in patients with LDH (19, 20). Nevertheless, exercise-based interventions alone may have limited effects on soft tissue relaxation and pain modulation, particularly in the early stages of treatment. Given their distinct yet complementary mechanisms, Tuina therapy and core stability training may exert synergistic effects in the management of LDH. Tuina primarily improves soft tissue condition and alleviates pain, whereas core stability training enhances neuromuscular control and spinal stability. However, current evidence remains insufficient to determine whether the combined intervention is more effective than either therapy alone, particularly in improving lumbar stability and related clinical outcomes.

Consequently, the present study proposes to conduct a randomized controlled clinical trial with blinded outcome assessment and data analysis to further validate the effectiveness of the combined intervention. A combination of musculoskeletal ultrasound, imaging assessments, pressure biofeedback unit assessment, and patient-reported outcome measures will be used to comprehensively evaluate lumbar stability and clinical outcomes. This approach will help determine whether the combined therapy provides superior benefits compared with single interventions.

## 2 Methods

### 2.1 Study design

**Figure 1:**
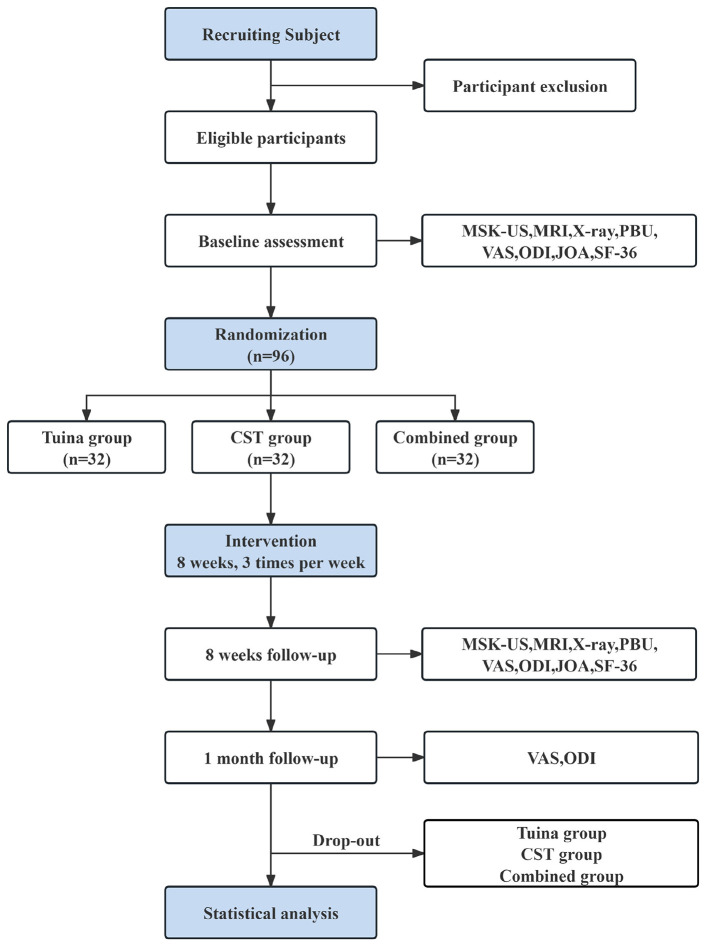
The flow chart of this trial.

This study is a single-center, three-arm parallel explanatory randomized controlled trial conducted in China (Figure 1). A total of 96 patients with LDH will be recruited and randomly allocated in a 1:1:1 ratio to the Tuina group, the core stability training group, or the combined intervention group. Participants who meet the inclusion criteria will be enrolled after providing written informed consent. Participants will be recruited from 15 October 2025 to 1 November 2027 through outpatient clinics, public advertisements, and social media platforms. All three groups will receive an 8-week intervention, administered three times per week, followed by a 1-month follow-up period. Outcome assessments will be conducted at baseline, after 8 weeks of intervention, and at the end of the follow-up period. The intervention duration was determined based on dose–response evidence, direct clinical trials, and prior rehabilitation literature. A recent systematic review and meta-analysis on stabilization-based exercise in patients with chronic low back pain reported that 8–12-week interventions produced the greatest effects on pain reduction (SMD = –0.88) and disability improvement (SMD = –0.85), with high-certainty evidence for disability outcomes (21). Consistent with this evidence, randomized controlled trials in patients with LDH have commonly adopted 8-week core stabilization protocols. An 8-week motor control training program significantly improved pain, disability, and transversus abdominis activation (22), while an 8-week program (three sessions per week) also demonstrated significant improvements in pain, disability, and trunk muscle endurance (23). Accordingly, an 8-week intervention period was selected, representing a commonly adopted duration within the evidence-supported 8–12-week therapeutic window for stabilization-based interventions in LDH. A 1-month follow-up assessment was included to evaluate the short-term maintenance of treatment effects after completion of the intervention, which is consistent with early post-intervention assessment time points used in randomized controlled trials of LDH (24). To enhance methodological rigor and minimize potential bias, blinded outcome assessment and statistical analysis procedures were incorporated into the study design. Outcome assessment and statistical analysis will be performed by two independent researchers who are blinded to group allocation. The primary and secondary outcome measures include musculoskeletal ultrasound, imaging assessments, pressure biofeedback unit assessment, and patient-reported clinical outcomes to comprehensively assess lumbar stability, pain, and functional status. The study protocol was developed in accordance with the SPIRIT guidelines, and the completed SPIRIT checklist is provided as Supplementary Table S1. In addition, a treatment fidelity checklist is provided as Supplementary Table S2 to ensure consistency and quality of intervention delivery.

### 2.2 Participants

#### 2.2.1 Inclusion criteria

1. Diagnosis of lumbar disc herniation (LDH) according to established diagnostic criteria (25), confirmed by MRI showing disc protrusion, extrusion, or sequestration at L3/4, L4/5, or L5/S1 levels. Mild-to-moderate degenerative changes (Pfirrmann grade ≤ III) in the absence of significant nerve root compression do not exclude participation.
2. Age ≥ 20 and ≤ 55 years, regardless of sex (3, 26).
3. Duration of the disease ≥ 3 months (27, 28).
4. Visual Analog Scale (VAS) score ≥ 3 and ≤ 6 (29).
5. Provision of written informed consent, voluntary agreement to participate, and ability to comply with the treatment protocol.

#### 2.2.2 Exclusion criteria

1. Presence of other serious diseases, such as tumors, fractures, infections, or tuberculosis.
2. Spinal deformities, severe spinal trauma, or a history of spinal surgery.
3. Severe osteoporosis (T-score ≤ –2.5), ankylosing spondylitis, or acute vertebral fracture.
4. Women who are pregnant or breastfeeding.
5. Individuals with psychiatric disorders, significant physical disabilities, or cognitive impairments.
6. Current or anticipated receipt of any concomitant treatments (e.g., medication, surgery, or alternative rehabilitation therapies) during the study period.

#### 2.2.3 Withdrawal criteria

1. Failure to complete the treatment according to the study protocol after enrollment.
2. Poor compliance during the trial, including modification of the treatment protocol or receipt of other related therapies.
3. Withdrawal from the study at the participant's request or termination of participation during the trial.

**Note:** Participants who withdraw from the intervention will still be encouraged to complete follow-up assessments whenever possible. All randomized participants will be included in the intention-to-treat analysis regardless of adherence.

#### 2.2.4 Termination criteria

1. The participant experiences serious adverse events related to the treatment intervention.
2. Severe complications or significant deterioration of the disease occur during the study period.
3. The investigator determines that the participant meets the criteria for trial termination according to the standard operating procedures, and the intervention should be stopped immediately.

### 2.3 Randomization

Participants will be randomly assigned in a 1:1:1 ratio to three groups in this single-center trial. A total of 96 patients with LDH who met the eligibility criteria will be enrolled and randomly allocated in a 1:1:1 ratio to the Tuina group, the core stability training group, or the combined Tuina plus core stability training group. The randomization sequence will be generated by an independent researcher using SPSS statistical software (version 27.0.1; IBM Corp., Armonk, NY, USA). To ensure allocation concealment, the random sequence numbers will be placed in sequentially numbered, opaque, sealed envelopes. Participants who meet the inclusion criteria and provide written informed consent will receive the envelopes in the order of recruitment, and the envelopes will be opened prior to the intervention to determine group assignment.

### 2.4 Blinding

In this study, blinding will be implemented for outcome assessors and data analysts. Group allocation will be coded to ensure objectivity during the evaluation and analysis processes. Due to the nature of the interventions, blinding of therapists and participants will not be feasible.

#### 2.4.1 Objective outcomes

(Musculoskeletal ultrasound, MRI, X-ray, and pressure biofeedback unit) will be assessed by independent researchers who are blinded to group allocation and not involved in the intervention delivery.

#### 2.4.2 Patient-reported outcomes

(Visual Analog Scale, Oswestry Disability Index, Japanese Orthopedic Association score, and 36-Item Short Form Health Survey) are inherently subjective and cannot be blinded, as participants are aware of their treatment assignment. Therefore, these outcomes are at risk of detection bias, which will be acknowledged as a limitation. Statistical analyses will be performed without knowledge of the actual group assignments, and unblinding will occur only after completion of all statistical analyses. Premature unblinding will not be permitted unless serious adverse events or other emergencies occur.

### 2.5 Sample size calculation

The sample size was calculated using G*Power 3.1. The calculation was based on the primary outcome, namely the muscle activation ratio of the transversus abdominis assessed by musculoskeletal ultrasound. Previous studies in patients with low back pain have reported medium-to-large effect sizes (Cohen's d ≈ 0.71) for changes in transversus abdominis activation following core stability training (30). Furthermore, ultrasound studies in patients with LDH have demonstrated significant alterations in core muscle morphology (31), suggesting that similar or larger effects may be anticipated in this population. Therefore, a conservative effect size of 0.78 was assumed for the present study.The selected effect size was based on previous studies reporting moderate-to-large effects in similar populations and was conservatively estimated.

With a two-sided α of 0.05 and a power (1−β) of 0.80, the required sample size was 27 participants per group. Considering a 15% dropout rate, the final sample size was set at 96 participants, with 32 participants in each group.

### 2.6 Interventions

All interventions in this study will be administered by professionally trained therapists. Tuina therapy will be performed by experienced Tuina practitioners, while core stability training will be guided by senior rehabilitation therapists. To ensure consistency of the interventions, both therapists will receive standardized training and pass a clinical trial operation assessment prior to the initiation of the study. All participants will receive individualized treatment three times per week (once every other day) for a total duration of 8 weeks. During the trial period, participants will be requested to refrain from receiving other treatments related to the study interventions, such as medications, acupuncture, or physical therapy. Participants who receive prohibited concomitant treatments will be withdrawn from the intervention but retained for intention-to-treat analysis. If additional treatments are deemed medically necessary, the trial will be terminated for that participant, and the relevant information will be documented in the case report form (CRF).

#### 2.6.1 Tuina group

**Figure 2:**
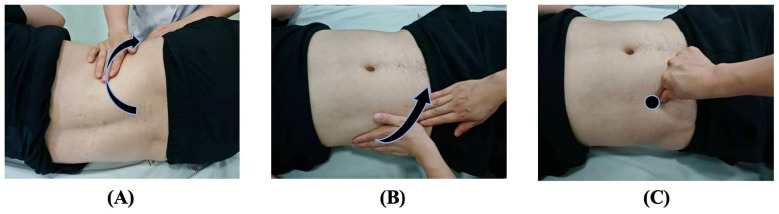
Tuina techniques. **(A)** Rubbing; **(B)** Pushing; **(C)** Pressing.

**Figure 3:**
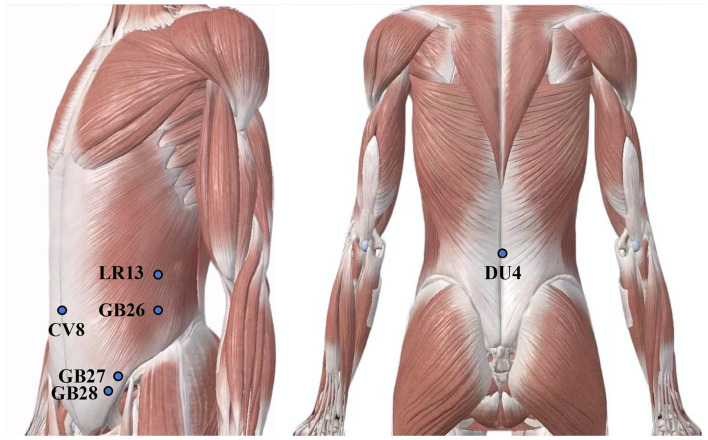
Locations of the acupoints. DU4 (Mingmen), LR13 (Zhangmen), GB26 (Daimai), CV8 (Shenque), GB27 (Wushu), and GB28 (Weidao).

The Tuina intervention comprised three standardized procedures targeting the lumbar–abdominal region. Firstly, all participants received standardized lumbar spine health education prior to the intervention. Subsequently, the Tuina procedures were performed with the participant in a supine position, with the hips and knees flexed.The procedures were carried out in the order of A–C (Figure 2), including rubbing, pushing, and acupressure techniques applied at predefined acupoints, namely Mingmen (DU4), Zhangmen (LR13), Daimai (GB26), Shenque (CV8), Wushu (GB27), and Weidao (GB28). The Tuina techniques and acupoint selection were based on a standardized national textbook (32). Each procedure was performed for approximately 3–6 min, and the three procedures constituted one complete session, lasting about 20 min. The manipulation intensity was standardized to achieve moderate pressure, defined as patient tolerance without pain. All procedures were conducted by trained practitioners. Treatments were administered three times per week for 8 weeks. The anatomical locations of the acupoints are shown in (Figure 3).

#### 2.6.2 Core stability training group

**Figure 4:**
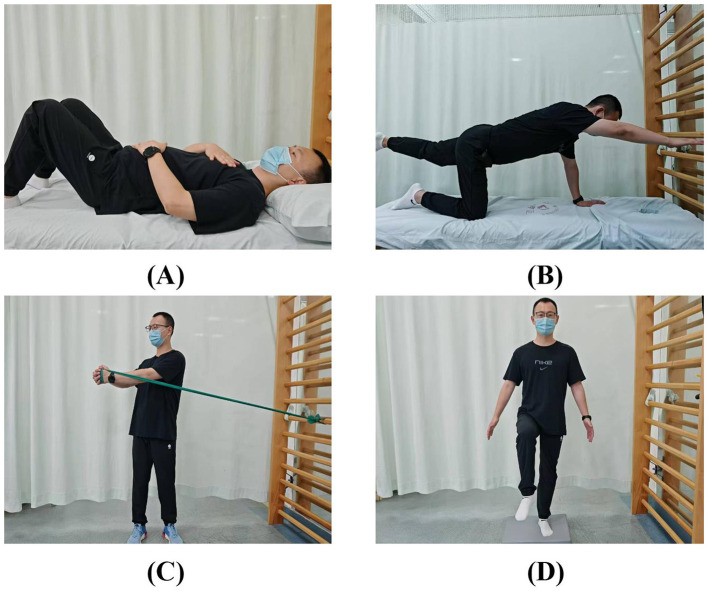
Core stability training procedures. **(A)** Diaphragmatic breathing; **(B)** Bird-dog exercise; **(C)** Trunk rotation; **(D)** Standing on a balance pad.

The CST protocol was developed based on established core stabilization principles and evidence-based recommendations for the management of chronic low back pain and LDH (6, 33). Specifically, the program was designed to progress from diaphragmatic breathing to quadruped contralateral limb extension and standing anti-rotation exercises. The exercise progression was informed by evidence supporting the role of breathing control, deep trunk muscle activation, and progressively challenging stabilization tasks in core stability training (34–36). The selected exercises were intended to facilitate coordinated activation of the transversus abdominis, multifidus, and related trunk musculature while maintaining safety and feasibility for patients with LDH. Firstly, all participants received standardized lumbar spine health education prior to the intervention. Subsequently, the exercises were performed under the supervision of a rehabilitation therapist. The exercises were carried out in the order of A–D (Figure 4), including diaphragmatic breathing training in the supine position, quadruped contralateral limb extension (bird-dog exercise), standing trunk rotation with elastic resistance, and balance training on an unstable surface. Each exercise was performed for three sets, with 10–15 repetitions or maintained for 6–10 s or approximately 1 min, depending on the specific task. A rest interval of 30–60 s was provided between sets and exercises to minimize fatigue. The total duration of each training session was approximately 20 min. Exercise intensity was adjusted according to participant tolerance to maintain a moderate level of exertion without exacerbating pain. Treatments were administered three times per week for 8 weeks.

#### 2.6.3 Combined Tuina and core stability training group

Firstly, all participants received the same standardized lumbar spine health education as described for the other two groups. Subsequently, the combined intervention consisted of sequential Tuina therapy followed by core stability training, as described above. The Tuina procedure was performed first, followed immediately by the core stability training within the same session. The total duration of each session was approximately 40 min. Treatments were administered three times per week for 8 weeks. The difference in intervention duration between groups will be treated as a potential confounding factor and will be included as a covariate in the linear mixed-effects models to control for its potential influence on the outcomes.

### 2.7 Outcomes

**Figure 5:**
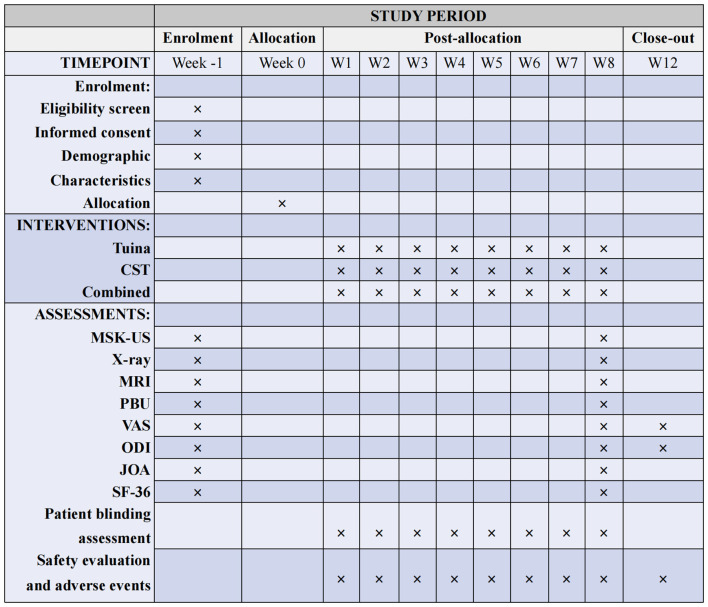
SPIRIT schedule. Enrollment, Intervention, and Assessment Schedule. CST, Core stability training group; MSK-US, Musculoskeletal ultrasound; MRI, Magnetic Resonance Imaging; VAS, Visual Analog Scale; ODI, Oswestry Disability Index; JOA, Japanese Orthopedic Association; SF-36, Short Form-36 Health Survey; PBU, Pressure Biofeedback Unit.

The efficacy evaluation in this study will focus on the primary and secondary outcome measures assessed at baseline and immediately after the intervention. In addition, selected clinical outcomes, including pain intensity and functional status, will be reassessed at the 1-month follow-up using the VAS and ODI to evaluate the persistence of treatment effects. Follow-up assessments will be conducted by independent outcome assessors who are blinded to group allocation.All outcome assessments will be conducted by independent researchers who are blinded to group allocation and are not involved in other aspects of the study. The schedule of enrolment, interventions, and assessments is presented in (Figure 5).

#### 2.7.1 Primary outcome

The primary outcome is the muscle activation ratio of the transversus abdominis (TrA) assessed by musculoskeletal ultrasound, which reflects the neuromuscular control capacity of the deep core musculature. The muscle activation ratio is calculated as follows:


$$\begin{array}{l} \text{Activation ratio}=\frac{\text{muscle thickness duringcontraction}}{\text{muscle thickness at rest}} \end{array}$$

This measure has been widely used as an indicator of deep core muscle activation.

##### 2.7.1.1 Musculoskeletal ultrasound assessment

Before and after the intervention, musculoskeletal ultrasound examinations will be performed using a Siemens Sequoia Silver ultrasound system (probe frequency: 5–12 MHz). The external oblique (EO), internal oblique (IO), transversus abdominis (TrA), and lumbar multifidus muscle (LMM) will be assessed in both groups. For EO, IO, and TrA measurements, the probe will be positioned at the level of the umbilicus along the mid-axillary line. The LMM will be located approximately 2 cm lateral to the L4 spinous process. Muscle thickness will be measured under standardized tasks: active straight-leg raising of the right lower limb in the supine position will be used for EO, IO, and TrA assessment, while single-leg standing on the left lower limb in the upright position will be used for LMM assessment. For each muscle, thickness will be measured three times at rest, at the end of standardized inspiration, and at the end of standardized expiration, and the mean value will be recorded. The muscle activation ratio will be calculated as the ratio of muscle thickness during contraction to that at rest. All measurements will be performed by an experienced sonographer. A subset of measurements will be repeated to assess intra-rater reliability.

#### 2.7.2 Secondary outcomes

Secondary outcomes include imaging parameters, functional assessments, and patient-reported clinical outcomes, which are used to comprehensively evaluate lumbar stability, pain, and functional status.

##### 2.7.2.1 Imaging assessments

Magnetic resonance imaging (MRI) and X-ray examinations will be performed before and after the intervention to evaluate structural and functional changes of the lumbar spine. MRI scans will be conducted using a 3.0-T system to obtain axial T2-weighted images at the L4/5 level. The cross-sectional area (CSA), functional cross-sectional area (FCSA), and fatty infiltration of the lumbar multifidus muscles will be measured. Intervertebral disc degeneration will be assessed using the Pfirrmann grading system.

Standing flexion–extension lateral radiographs will be obtained to measure sagittal translation (ST), segmental angulation (SA), lumbosacral angle (LSA), and lumbar lordosis (Cobb angle from L1 to S1), which are used to evaluate lumbar stability. All imaging measurements will be performed independently by experienced radiologists and repeated three times, with the mean value recorded.

##### 2.7.2.2 Pressure biofeedback unit (PBU) assessment

The pressure biofeedback unit (PBU) is used to assess deep core muscle function by monitoring pressure changes (37). Participants will perform standardized abdominal contraction tasks in the prone position. A mean pressure decrease of ≥ 4 mmHg across three trials will be considered indicative of effective transversus abdominis activation.

##### 2.7.2.3 Patient-reported outcomes

Pain intensity will be assessed using the visual analog scale (VAS), ranging from 0 to 10, with higher scores indicating greater pain (38).

Functional disability will be evaluated using the Oswestry Disability Index (ODI), with scores ranging from 0% to 100%, where higher scores indicate greater disability (39).

Lumbar function will be assessed using the Japanese Orthopedic Association (JOA) score, with a maximum score of 29 and higher scores indicating better function (40).

Health-related quality of life will be evaluated using the 36-Item Short Form Health Survey (SF-36), with scores ranging from 0 to 100 for each domain and higher scores indicating better health status (41).

All outcome data will be recorded and verified by independent researchers. The collected data will be used for within-group comparisons before and after treatment, as well as between-group comparisons.Symptom recurrence will be defined as a clinically meaningful increase in pain intensity or functional disability compared with post-intervention levels, exceeding the minimal clinically important difference (MCID), where applicable.

#### 2.7.3 Adverse events

All adverse events occurring during the intervention and follow-up period will be recorded and monitored, including but not limited to: increased local pain, muscle soreness, fatigue, skin abrasion or ecchymosis from Tuina, and exacerbation of radicular symptoms. Any discomfort or unexpected events will be documented in detail, including their type, severity, duration, and management. The relationship between adverse events and the intervention will be evaluated. Serious adverse events will be reported immediately to the ethics committee, and appropriate medical management will be provided. If necessary, the trial will be terminated to ensure participant safety.

### 2.8 Quality control

#### 2.8.1 Intervention delivery and fidelity

All interventions will be delivered by licensed practitioners with at least 3 years of clinical experience. Prior to trial initiation, all practitioners will undergo standardized training and pass a clinical trial operation assessment to ensure consistency in intervention delivery, including treatment intensity, frequency, and procedures according to the study protocol.

All Tuina treatments will be delivered by the same licensed Tuina practitioner, and all core stability training sessions will be supervised by the same licensed rehabilitation therapist throughout the study. Although therapist blinding is not feasible due to the nature of the interventions, therapists will strictly follow standardized treatment manuals and will not be involved in outcome assessment, data collection, or statistical analysis.

To ensure protocol adherence and minimize variability in treatment delivery, regular supervision sessions will be conducted throughout the study. Treatment fidelity will be monitored using a standardized checklist (Supplementary Table S2) and periodic supervision. In addition, a random sample of treatment sessions will be independently observed or recorded for fidelity evaluation. Adherence to the intervention protocol will also be documented throughout the study.

#### 2.8.2 Outcome assessment

All outcome assessors will receive standardized training prior to study initiation to ensure consistency in data collection and scoring criteria. Whenever possible, assessments for the same participant will be conducted by the same assessor to minimize observer bias. Case report forms (CRFs) will be completed accurately and in full, without unauthorized modifications.

#### 2.8.3 Participant management

Before the intervention, each participant will receive detailed instructions regarding the study procedures, precautions, and protocol requirements, and will be advised to refrain from using other interventions during the study period. Throughout the study, the research team will provide health consultation and monitoring. If pain or discomfort occurs during the intervention, participants will be instructed to promptly inform the research team, and appropriate medical management will be provided as necessary. Any adverse events or unexpected incidents will be recorded in detail, including their type, time of occurrence, severity, duration, and management measures.

### 2.9 Data monitoring

Given the low-risk nature of the interventions in this study, no formal data monitoring committee (DMC) will be established. The study will be supervised by the research team of the Affiliated Rehabilitation Hospital of Fujian University of Traditional Chinese Medicine. All adverse events will be continuously monitored, recorded, and reviewed to ensure participant safety. Any serious adverse events will be reported promptly to the ethics committee, and appropriate measures will be taken if necessary.

### 2.10 Data collection and management

A paper-based case report form (CRF) will be used for data collection. Outcome assessors will complete all CRFs in accordance with the study protocol, including treatment efficacy, adverse events, questionnaire data, musculoskeletal ultrasound results, and lumbar imaging findings. All data will be recorded accurately and comprehensively, with minimal corrections.The screening phase will last 1 week and will include the collection of baseline information, such as chief complaints, history of present illness, past medical history, family history, and routine physical examination findings. Any values outside clinically acceptable ranges will be reviewed and justified.All completed CRFs will be reviewed by designated research personnel before submission for data entry. Data will be entered into a Microsoft Excel database by two independent data managers who are not involved in the study, and double data entry and cross-checking will be performed to ensure accuracy and consistency with the original source documents. In case of discrepancies or uncertainties in the recorded data, queries will be resolved through consultation with the responsible investigators, and corrections will be made accordingly. After data verification, the final database will be confirmed and locked prior to statistical analysis. The study process will be supervised and audited by the Affiliated Rehabilitation Hospital of Fujian University of Traditional Chinese Medicine. All participant data will be kept confidential and will not be disclosed without permission.

### 2.11 Statistical analysis

All statistical analyses will be performed using IBM SPSS Statistics (Version 27.0). Efficacy analyses will follow the intention-to-treat (ITT) principle, including all randomized participants. Continuous variables will be tested for normality and presented as mean ± standard deviation or median (interquartile range), as appropriate, while categorical variables will be expressed as counts and percentages. Baseline characteristics will be summarized descriptively without formal statistical comparisons. Primary and secondary outcomes will be analyzed using linear mixed-effects models, with group, time, and group-by-time interaction as fixed effects and subjects as random effects. Intervention duration (20 vs. 40 min) and baseline values of the outcome measures will be explicitly included as covariates to account for potential confounding effects. Therapist identity will be included as a random intercept to account for potential clustering effects. Model assumptions will be evaluated using residual diagnostics. If significant group-by-time interactions are detected, post-hoc pairwise comparisons with Bonferroni correction will be performed. Missing data will be handled using multiple imputation under the missing-at-random assumption. Safety analyses will include all participants who received at least one treatment, and adverse events will be compared using the chi-square test or Fisher's exact test, as appropriate. Sensitivity analyses, including complete-case analyses, per-protocol analyses, and analyses adjusted for intervention duration, will be conducted to assess the robustness of the primary outcome. All statistical tests will be two-sided, with a significance level set at *P* < 0.05, and 95% confidence intervals will be reported. Effect sizes (e.g., Cohen's d) will also be reported to facilitate interpretation of the clinical relevance of the findings.

## 3 Discussion

To our knowledge, this study is among the first randomized controlled trials to investigate the combined effects of Tuina and core stability training in patients with lumbar disc herniation (LDH). Although both interventions are commonly used in clinical practice, high-quality evidence evaluating their combined efficacy remains limited. Given the high prevalence, prolonged disease course, and increasing trend toward younger onset of LDH, there is a need to explore effective and evidence-based rehabilitation strategies (25, 42). Tuina and core stability training may exert complementary effects, with Tuina primarily improving soft tissue mobility and pain, and core stability training enhancing neuromuscular control and spinal stability (18, 43). The combination may therefore provide complementary and potentially synergistic effects. This integrative approach may address both passive tissue dysfunction and active neuromuscular deficits.

This study has several strengths. It employs a randomized controlled design with blinded outcome assessment and data analysis to minimize potential bias. In addition, multiple outcome measures, including musculoskeletal ultrasound, lumbar imaging, and clinical assessments, are incorporated to comprehensively evaluate changes in lumbar stability and function. Importantly, this study focuses on lumbar stability as a core outcome, which has been insufficiently addressed in previous studies. In addition, the inclusion of a follow-up period allows for the assessment of short-term sustainability of treatment effects.

However, several limitations should be acknowledged. This is a single-center study with a relatively small sample size, which may limit generalizability. Blinding of participants and therapists was not feasible due to the nature of the interventions, which may introduce performance bias and expectation effects. Specifically, patient-reported outcomes (VAS, ODI, JOA, and SF-36) may be influenced by participants' perceptions and treatment expectations. To mitigate these biases, objective outcome measures—including musculoskeletal ultrasound, MRI, X-ray, and pressure biofeedback—will be assessed by independent evaluators blinded to group allocation, and statistical analyses will be conducted by investigators blinded to group allocation. Certain measurements, particularly ultrasound assessments, may still be subject to operator-dependent variability (44). Some mechanistic inferences rely on prior literature rather than direct measurement, and the absence of a sham or placebo control may further introduce expectation-related effects. The combined intervention group received twice the total intervention duration compared to monotherapy groups (20 vs. 40 min per session), although intervention duration will be included as a covariate, residual confounding cannot be entirely excluded. The upper age limit of 55 years restricts generalizability to older adults with LDH, and despite standardized training, some operator-dependent variability in Tuina procedures may remain. Future multicenter studies with larger sample sizes, longer follow-up periods, and time-matched control designs are warranted to confirm these findings and isolate the specific effects of the combined intervention. Overall, this study is expected to provide preliminary evidence and methodological guidance for integrating Tuina and core stability training in LDH management.

If proven effective, this combined approach may provide a practical and scalable rehabilitation strategy for patients with LDH in clinical settings.
