# Supplementary material for: Efficacy of Tuina combined with core stability training on lumbar stability and clinical outcomes in patients with lumbar disc herniation: a randomized controlled trial protocol
Source: Front Med (Lausanne). 2026 Jul 7;13:1857731. doi: 10.3389/fmed.2026.1857731 (PMC13339916; doi:10.3389/fmed.2026.1857731)
Supplement: Supplementary file 1 [file Table_1.docx]

# Supplementary Table S2. Treatment Fidelity Checklist

| Domain | Item | Description | Planned | Monitoring Method |
| --- | --- | --- | --- | --- |
| Study design | Standardized intervention protocol | All interventions follow predefined procedures | Yes | Protocol document |
| Study design | Treatment dose consistency | 3 sessions/week for 8 weeks | Yes | CRF records |
| Training | Therapist qualification | Licensed practitioners with ≥3 years experience | Yes | Certification verification |
| Training | Standardized training | Unified training before trial | Yes | Training records |
| Training | Competency assessment | Must pass assessment before participation | Yes | Assessment documentation |
| Delivery | Intervention standardization | Tuina and CST follow predefined procedures | Yes | Supervision/checklist |
| Delivery | Treatment duration control | Tuina ~20 min, CST ~20 min, combined ~40 min | Yes | Session records |
| Delivery | Intensity control | Moderate intensity without pain | Yes | Therapist report |
| Delivery | Individualization | Adjusted based on tolerance without protocol deviation | Yes | CRF notes |
| Fidelity | Fidelity monitoring | Assessed using standardized checklist | Yes | Checklist |
| Fidelity | Supervision | Regular supervision | Yes | Monitoring logs |
| Fidelity | Random audit | Random sessions observed/recorded | Yes | Audit records |
| Adherence | Attendance monitoring | All sessions recorded | Yes | Attendance logs |
| Adherence | Protocol compliance | Avoid other treatments | Yes | Self-report + CRF |
| Adherence | Deviation recording | Additional treatments documented | Yes | CRF documentation |
| Receipt | Participant instruction | Standardized health education provided | Yes | Education records |
| Receipt | Understanding | Participants informed of procedures | Yes | Verbal confirmation |
| Data quality | Outcome assessor training | Assessors trained | Yes | Training records |
| Data quality | Blinding | Assessors blinded | Yes | Protocol |
| Data quality | Data entry control | Double entry and cross-check | Yes | Database logs |
