# Supplementary material for: Efficacy of Tuina combined with core stability training on lumbar stability and clinical outcomes in patients with lumbar disc herniation: a randomized controlled trial protocol
Source: Front Med (Lausanne). 2026 Jul 7;13:1857731. doi: 10.3389/fmed.2026.1857731 (PMC13339916; doi:10.3389/fmed.2026.1857731)
Supplement: Supplementary file 2 [file Table_2.docx]

**S1 File. SPIRIT Checklist**

| Section | Item | Description |
| --- | --- | --- |
| Title | 1 | Effect of Tuina combined with core stability training on lumbar stability and clinical outcomes in patients with lumbar disc herniation: Study protocol for a randomized controlled trial |
| Trial Registration | 2a | Prospective registration in International Traditional Medicine Clinical Trial Registry (ITMCTR), number ITMCTR2025001794 |
| Protocol Version | 3 | Version 1.0, March 2026 |
| Funding | 4 | Supported by Fujian Provincial Natural Science Foundation (2023J06037) |
| Roles and Responsibilities | 5 | Principal Investigator, Co-Investigators, Data Managers, Outcome Assessors, Rehabilitation Therapists |
| Background and Rationale | 6 | LDH has a high incidence, prolonged course, and increasingly younger onset. Tuina combined with core stability training may improve lumbar stability and reduce pain more effectively than conventional rehabilitation. Tuina aims to release fascia adhesions, correct vertebral alignment, and relieve nerve compression; core stability training activates deep trunk muscles. |
| Objectives | 7 | To evaluate the efficacy and safety of Tuina combined with core stability training in improving lumbar stability and clinical outcomes in LDH patients |
| Trial Design | 8 | Randomized controlled trial with three parallel groups: Tuina group, Core stability training group, Combined group |
| Eligibility Criteria – Inclusion | 9 | 1. Diagnosis of lumbar disc herniation (LDH) according to established diagnostic criteria (Huang et al., 2023). 2. Age between 20 and 55 years. 3. MRI-confirmed LDH with nerve root compression corresponding to clinical symptoms, with at least one objective neurological sign. 4. Visual Analog Scale (VAS) score between 3 and 6. 5. Provided written informed consent, voluntarily agreed to participate in the study, and able to comply with the treatment protocol. |
| Eligibility Criteria – Exclusion | 10 | 1. Presence of other serious diseases, such as tumors, fractures, infections, or tuberculosis. 2. Spinal deformities, severe spinal trauma, or a history of spinal surgery. 3. Women who are pregnant or breastfeeding. 4. Individuals with psychiatric disorders, significant physical disabilities, or cognitive impairments. 5. Patients who are currently receiving or expected to receive any concomitant treatments (e.g., medication, surgery, or alternative rehabilitation therapies) during the study period. |
| Discontinuation Criteria | 11 | 1. Failure to complete protocol; 2. Poor compliance; 2. Withdrawal; 3. Serious adverse events or deterioration |
| Interventions – Tuina | 12 | Supine position; rubbing DU4→LR13→GB26→CV8 (3 min); pushing along iliac crest (3 min); acupressure GB26/27/28 (2 min each side); total 20 min |
| Interventions – Core Stability | 13 | Diaphragmatic breathing (10×3); bird-dog (10 reps/side); trunk rotation (10–15 reps); balance training (~1 min each) |
| Combined Group | 14 | Combination of Tuina and core stability training |
| Primary Outcome | 15 | Musculoskeletal ultrasound assessment |
| Secondary Outcomes | 16 | MRI,X-ray,PBU,VAS,ODI,JOA ,SF-36. |
| Safety Monitoring | 17 | Adverse events recorded in CRF; severity and causality assessed; reported to ethics committee |
| Participant Timeline | 18 | Screening, baseline, intervention, post-treatment, 1-month follow-up |
| Sample Size | 19 | Calculated using GPower (effect size 0.78, α=0.05, power=0.80); 27 per group; 32 per group after 15% dropout; total 96. |
| Data Collection and Management | 20 | CRFs completed by assessors; double data entry; database verification; missing data handled by multiple imputation |
| Statistical Analysis | 21 | t-test/ANOVA for normal data; Wilcoxon/Kruskal–Wallis for non-normal; chi-square for categorical; mixed-effects model for repeated measures; *P* < 0.05 |
| Quality Control – Outcome Assessment | 22 | Standardized training; same assessor when possible; CRF verification |
| Quality Control – Participants | 23 | Instructions before intervention; adherence monitoring; report discomfort promptly |
| Quality Control – Data Management | 24 | CRFs accurate and complete; database consistent with source documents |
| Ethics and Dissemination | 25 | Ethical approval obtained; informed consent will be obtained from all participants; findings will be disseminated according to institutional policies. |
